# Geographically Distinct Circulation of Genotype II and III St. Louis Encephalitis Virus, Texas, USA, 2009–2024

**DOI:** 10.3201/eid3204.250934

**Published:** 2026-04

**Authors:** Alexander R. Kneubehl, Daniel P. Rehm, Michael W. Curtis, Bianca M. Wimmer, Bethany Bolling, Angie Broussard, Jeremy Vela, Jennifer Rocha, Lindsey Templeton, Maximea Vigilant, Courtney Standlee, Steven M. Presley, Job E. Lopez, Shannon E. Ronca

**Affiliations:** Baylor College of Medicine, Houston, Texas, USA (A.R. Kneubehl, D.P. Rehm, M.W. Curtis, J.E. Lopez, S.E. Ronca); Texas Children’s Hospital William T. Schearer Center for Human Immunobiology, Houston (A.R. Kneubehl, D.P. Rehm, S.E. Ronca); Texas Tech University Institute of Environmental and Human Health, Lubbock, Texas, USA (B.M. Wimmer, S.M. Presley); Texas Department of State Health Services, Austin, Texas, USA (B. Bolling); Harris County Public Health Division of Mosquito and Vector Control, Houston (A. Broussard, J. Vela, J. Rocha, L. Templeton, M. Vigilant, C. Standlee)

**Keywords:** St. Louis encephalitis virus, viruses, meningitis/encephalitis, genomics, surveillance, flavivirus, vector-borne infections, mosquito-borne infections, West Nile virus, Texas, United States

## Abstract

We conducted a retrospective genomic surveillance study of St. Louis encephalitis virus (SLEV) in Texas, USA, to determine the genotypes circulating in the region. By using a custom tiled-amplicon assay with Oxford Nanopore sequencing, we generated 63 genomes from SLEV-positive mosquito pools and viral isolates collected during 2009–2024. Phylogenomic analysis revealed temporal overlap of genotype II and III circulation, but with distinct geographic segregation. Genotype II was confined to Gulf Coast counties with sustained local transmission, whereas genotype III was only in north and west Texas, but with persistent circulation and repeated introductions. We identified the earliest known US genotype III sequences, although their phylogenetic placement leaves the entry point of genotype III into the United States unresolved. Our findings emphasize the need for clinical vigilance in West Texas, where SLEV and West Nile virus co-circulate, and suggest the Gulf Coast may be buffered against foreign genotype introduction.

St. Louis encephalitis virus (SLEV) is a recently reemerged mosquitoborne orthoflavivirus found in the Americas (*1*). SLEV is maintained in an enzootic cycle between mosquitoes (primarily *Culex* spp.) and avian reservoirs; mammals are dead-end hosts (*2*–*5*). Since the virus’s first identification in 1933 in St. Louis, Missouri, USA (*6*), focal SLEV outbreaks have been reported throughout the United States; disease manifestations range from nonspecific influenza-like symptoms to severe, potentially fatal, neurologic complications (*7*–*9*). After West Nile virus (WNV) was introduced to the United States in 1999 (*10*), a marked decrease in SLEV-positive mosquito pools and human cases occurred in areas where WNV was present (*11*,*12*). Both viruses, members of the Japanese encephalitis virus serocomplex, confer cross-reactive immunity in avian hosts (*13*–*15*), and the decrease in SLEV occurrence potentially is driven by ecologic competition between WNV and SLEV in reservoir populations because of cross-protective immune responses (*11*,*14*). SLEV reemerged in the United States after the introduction of a new genotype to the southwestern United States.

Eight genotypes of SLEV exist. Genotypes I, II, IV, and V are historically found in North America, and genotypes III, V, VI, VII, and VIII are found in South America (*16*,*17*). SLEV human cases and outbreaks have occurred in North America, and none had been reported in South America (*1*,*7*) until 2005, when an outbreak caused human fatalities in Argentina (*18*). Subsequent 2010 and 2011 outbreaks in Argentina were identified as genotype III, which previously had not been associated with human cases (*1*). In 2015, the first concurrent outbreak of WNV and SLEV occurred in Arizona, USA, prompting SLEV surveillance efforts (*19*,*20*). That same year, genotype III SLEV was detected in California, USA, after nearly a decade without reported activity despite ongoing monitoring. Retrospective analyses identified the viruses in both states as genotype III and revealed a 2014 mosquito pool from Arizona that was positive for SLEV and WNV (*20*). Genotype III outbreaks in California and Arizona had a case-fatality rate of ≈8% (*8*,*19*–*22*). Further genomic surveillance indicated that SLEV genotype III replaced all other SLEV genotypes in the western United States (*21*,*22*) but analyzed samples from as far east as El Paso, Texas, USA.

SLEV was a prevalent mosquitoborne virus in Texas, particularly along the Gulf Coast, before the introduction of WNV in 2002; SLEV cases and mosquito activity declined in the region after WNV was established (*1*,*23*–*26*). During the past 20 years, sporadic reports of SLEV human cases and SLEV-positive mosquito pools occurred throughout Texas (Figure 1, panels A–D). However, only 2 full-length Texas SLEV genomes (GenBank accession nos. EF158052.1 and MN233324.1) have been detected, and no information is available regarding which genotypes are currently circulating. To address those deficiencies and determine whether genotype III is widespread throughout Texas, we performed a retrospective genomic surveillance study analyzing SLEV genomes from SLEV-positive mosquito pools during 2009–2024.

**Figure 1:**
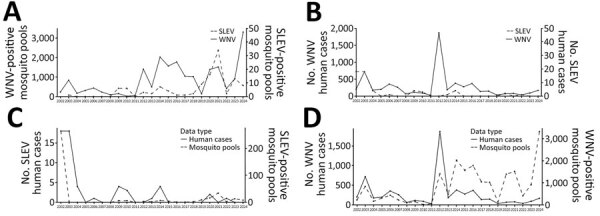
Annual SLEV and WNV mosquito and human case activity from study of circulation of genotype II and III SLEV, Texas, USA, 2002–2024. A) Annual number of reported SLEV-positive and WNV-positive mosquito pools. B) Annual number of reported human SLEV and WNV infection cases. C) Annual number of reported human SLEV infection cases and number of SLEV-positive mosquito pools. D) Annual number of reported human WNV infection cases and number of WNV-positive mosquito pools. Scales for the y-axes differ substantially to underscore patterns but do not permit direct comparisons. Data from Texas DSHS Arbovirus Activity Reports (https://www.dshs.texas.gov/mosquito-borne-diseases/dshs-arbovirus-weekly-activity-reports) and Texas DSHS Arbovirus Field Surveillance Report 1985–2012 (https://www.dshs.texas.gov/laboratory-services/programs-laboratories/microbiology-unit/arbovirus-surveillance-program/arbovirus-field-surveillance-report). SLEV, St. Louis encephalitis virus; WNV, West Nile virus.

## Materials and Methods

We have described in detail the methods used in this study (Appendix). In addition, a GitHub repository for this work (https://github.com/kneubehl/Texas-SLEV-Genome-Surveillance) (*27*) contains other ancillary items (e.g., scripts).

### Mosquito Pools

Mosquito pools were provided by the Texas Department of Safety and Health Services, Harris County Public Health Mosquito and Vector Control Division, or Texas Tech University’s Biologic Threat Research Laboratory, each having their own respective collection and testing strategies (Appendix). We summarized mosquito pool metadata (Appendix Table 1) (*27*). Because virus screening modalities varied on the basis of a mosquito pool’s origin, we screened all pools by using a real-time reverse transcription PCR (rRT-PCR) to confirm positivity.

### Virus Isolation

We performed virus isolation by incubating mosquito pool homogenate with Vero CCL81 cells in T75 flasks. When cytopathic effects were apparent or after 7 days, whichever came first, we clarified cell culture supernatants by using centrifugation. We stored an aliquot of supernatant in 2x DNA/RNA Shield (Zymo, https://www.zymoresearch.com) for RNA extraction. We obtained 4 SLEV isolates through BEI Resources at the National Institutes of Health National Institute of Allergy and Infectious Diseases (V07457, V08449, V08458, and TXAR 9-6038). We summarized virus isolate metadata (Appendix Table 2) (*27*).

### RNA Isolation and SLEV rRT-PCR Testing

We isolated total RNA and DNA from samples by using a modified Zymo Quick-DNA/RNA Pathogen Miniprep protocol (Appendix). We tested all samples for the presence of SLEV RNA by using a modified rRT-PCR protocol (*28*) (Appendix).

### Tiled-Amplicon Sequencing Primer Development

We designed a tiled-amplicon primer scheme to amplify the entire coding sequence of the SLEV genome (97.4% of the entire genome sequence) by using the Olivar tool with SLEV genomes sourced from GenBank. We used DQ525916.1 as the reference genome (*27*,*29*) (Appendix Table 3).

### Virus Genome Amplification, Sequencing, and Assembly

We generated SLEV tiled amplicons by using reverse transcription -PCR (Appendix). We prepared sequencing libraries by using the SQK-RBK114.96 kit (Oxford Nanopore Technologies, https://www.nanoporetech.com) and sequenced them with an R10.4.1 flow cell (Oxford Nanopore), per manufacturer’s instructions, on a MinION Mk1B (Oxford Nanopore) or PromethION P2 solo (Oxford Nanopore). We generated consensus genome assemblies by sample with the ViralRecon nextflow pipeline with the ARTIC minion pipeline (https://zenodo.org/records/7764938) option with modifications (Appendix). Sequencing data and genomes generated in this study are available through BioProject PRJNA1273178.

### Maximum-Likelihood Analyses of All SLEV Genotypes and SLEV Genotype II Envelope

To determine the phylogenetic placement of our SLEV assemblies, we inferred a maximum-likelihood tree of all SLEV genotypes with available genomes on GenBank. We obtained SLEV genomes from GenBank, and we analyzed only genomes with >70% completeness. We aligned the nucleotide sequences by using MAFFT (*30*). We inferred a maximum-likelihood tree by using IQ-TREE2 (*31*,*32*). We used the same process for SLEV genotype II envelope sequences. We visualized and annotated the phylogenies in R (The R Project for Statistical Computing, https://www.r-project.org) and Inkscape (Inkscape, https://inkscape.org). Newick tree files for each phylogeny are available (*27*).

### Bayesian Phylogenetic Analysis of SLEV Genotype III

We assessed the maximum-likelihood tree of all SLEV genotype III genomes with root-to-tip genetic divergence and time of sampling regression by using TempEst (*33*). That analysis determined a temporal signal that warranted generating a timetree (correlation coefficient 0.8284). We used BEAST for Bayesian phylogenetic analysis (*34*). We used an uncorrelated relaxed clock model, a general time reversible plus empirical base frequencies plus gamma-distributed rate heterogeneity with 4 categories substitution model, and a Bayesian skyline with a 2-groups tree prior and a Markov chain Monte Carlo chain length of 100 million with sampling every 10,000 trees to infer the timetree. We ran 2 independent MCMC chains, and we confirmed convergence with Tracer before combining with LogCombiner (*34*), using a 10% burn-in for each log and tree file. We generated the maximum clade credibility tree with TreeAnnotator (*34*). The XML generated by BEAUti for running BEAST and the nexus file generated by TreeAnnotator have been described previously (*27*). We visualized and annotated the phylogenies by using R and Inkscape.

## Results

### Genotyping SLEV-Positive *Cx. quinquefasciatus* and *Cx. tarsalis* Mosquito Pools 

**Figure 2:**
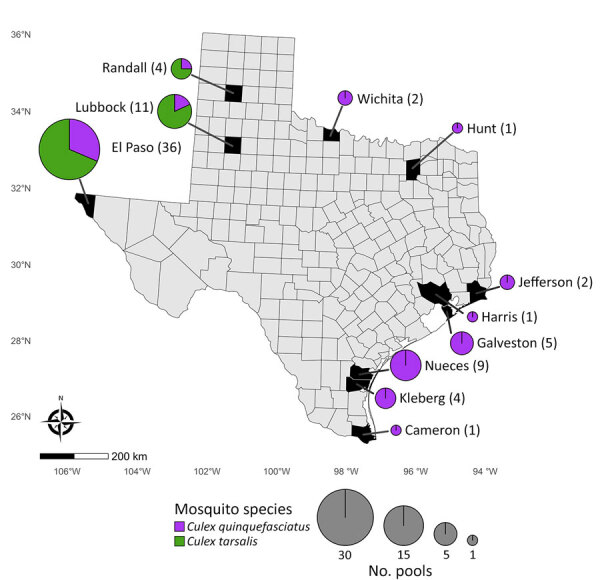
St. Louis encephalitis virus–positive mosquito pool samples from study of circulation of genotype II and III St. Louis encephalitis virus, by county of origin, Texas, USA, 2002–2024. Each county of origin is indicated in black. Pie charts indicate number of pools and proportion of *Culex* spp. mosquitoes contributing to those samples. Number of pools from each county is indicated in parentheses by the county name.

Our sample set included 75 SLEV-positive mosquito pools from 11 counties in Texas and 4 previously generated Texas SLEV isolates (Figure 2). Each pool consisted of *Cx. tarsalis* (36 pools) or *Cx. quinquefasciatus* (39 pools) collected during 2009–2024. *Cx. tarsalis* pools came from northwestern Texas, whereas *Cx. quinquefasciatus* pools were collected throughout the state (Figure 2). Our sample set included 5 mosquito pools positive for SLEV and WNV from El Paso County (4 pools) and Lubbock County (1 pool).

We used a tiled-amplicon sequencing approach for genome sequencing of SLEV. We also attempted virus isolation on all 64 mosquito pools not in a preservative to amplify the virus in case sequencing attempts from the mosquito pool RNA failed. Of the 123 samples tested (75 mosquito pools, 44 successful virus isolations, and 4 preexisting virus isolates), 63 unique SLEV genomes had >70% genome completeness. We assembled 37 genomes directly from mosquito pools, 22 from virus isolates that yielded more complete genomes than their corresponding mosquito pools, and 4 from previously available isolates. Six other mosquito pools had genome assemblies with <70% completeness; we genotyped those but did not use them for phylogenetic analysis. We plotted virus genome completeness with each sample’s cycle threshold (Ct) value determined by rRT-PCR for all 123 sequencing attempts (Appendix Figure 1) (*27*). The median Ct for samples that were successful (>70% genome completeness) was 22.7 (range 16.2–29.5). We summarized read depth across the genome from representative samples spanning different Ct values and genome recovery (Appendix Figure 2). We also summarized genome completeness for each sample (Appendix Tables 1, 2) (*27*).

### Phylogenetic Analysis of SLEV Genomes

**Figure 3:**
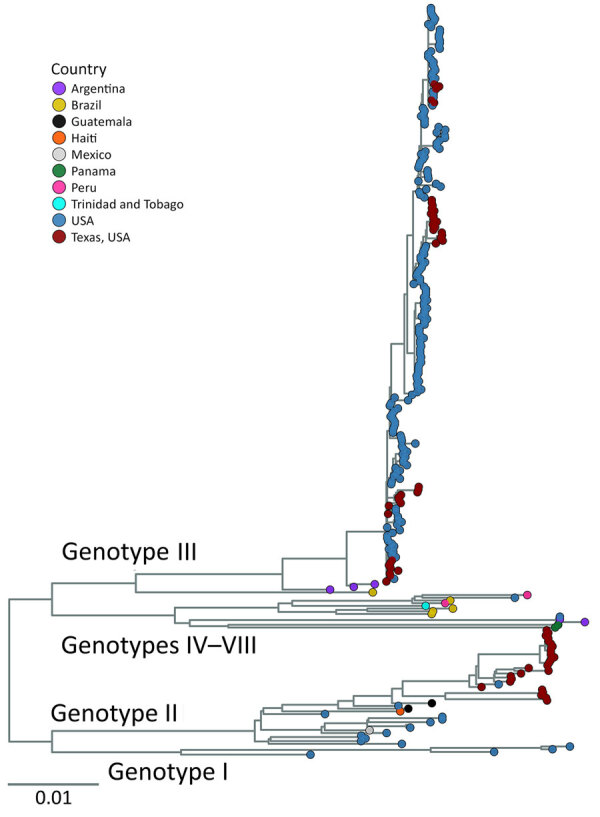
Maximum-likelihood phylogenic analysis of all St. Louis encephalitis virus genomes from study of circulation of genotype II and III St. Louis encephalitis virus, Texas, USA, 2009–2024. Maximum-likelihood inferred tree shows 276 genomes currently available that have <30% of the genome missing. Tip color represents sample’s country of origin; samples from Texas are labeled with a separate color (red) to distinguish Texas samples from rest of United States. Genotypes annotated on the tree indicate which clade contains which genotype or genotypes. Tree was midpoint rooted. Scale bar indicates number of nucleotide substitutions per site.

**Figure 4:**
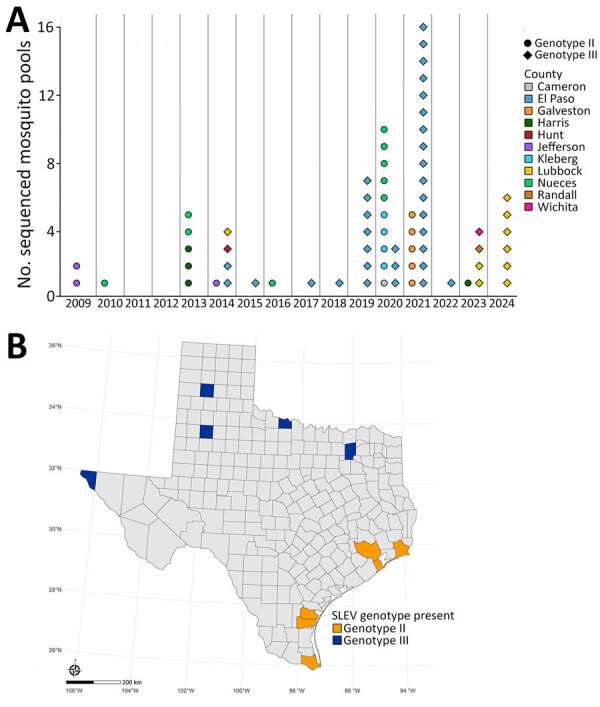
SLEV genotype distribution from study of circulation of genotype II and III SLEV, by year and county, Texas, USA, 2009–2024. A) Dot plot of number of sequenced SLEV-positive mosquito pools for each year, by genotype and county. Each shape represents an individual mosquito pool. B) Map of Texas indicating presence of SLEV genotype II or III. Each genotype exclusively found in its respective counties; no counties had presence of both genotypes. SLEV, St. Louis encephalitis virus.

Maximum-likelihood phylogenetic analyses defined the genotypes of our samples and their genetic relatedness to 276 previously published SLEV genomes (63 of our assemblies and 213 GenBank assemblies). We genotyped the 6 samples with a genome completeness <70% but >5% by using BLASTn (https://blast.ncbi.nlm.nih.gov) (Appendix Table 4) (*27*). Our analyses indicated that genotypes II (n = 26) and III (n = 43) were present in Texas concurrently (Figure 3). Genotype II was detected in 2009, 2010, 2013, 2014, 2016, 2020, 2021, and 2023; genotype III was present in 2014, 2015, and 2017–2024 (Figure 4, panel A). Although temporal overlap of genotypes occurred, we noted spatial segregation: genotype II in coastal counties (Cameron, Harris, Galveston, Jefferson, Kleberg, and Nueces) and genotype III in northern and western counties (El Paso, Hunt, Lubbock, Randall, and Wichita) (Figure 4, panel B).

### Phylogenetic Analysis of Genotype II Envelope Gene

**Figure 5:**
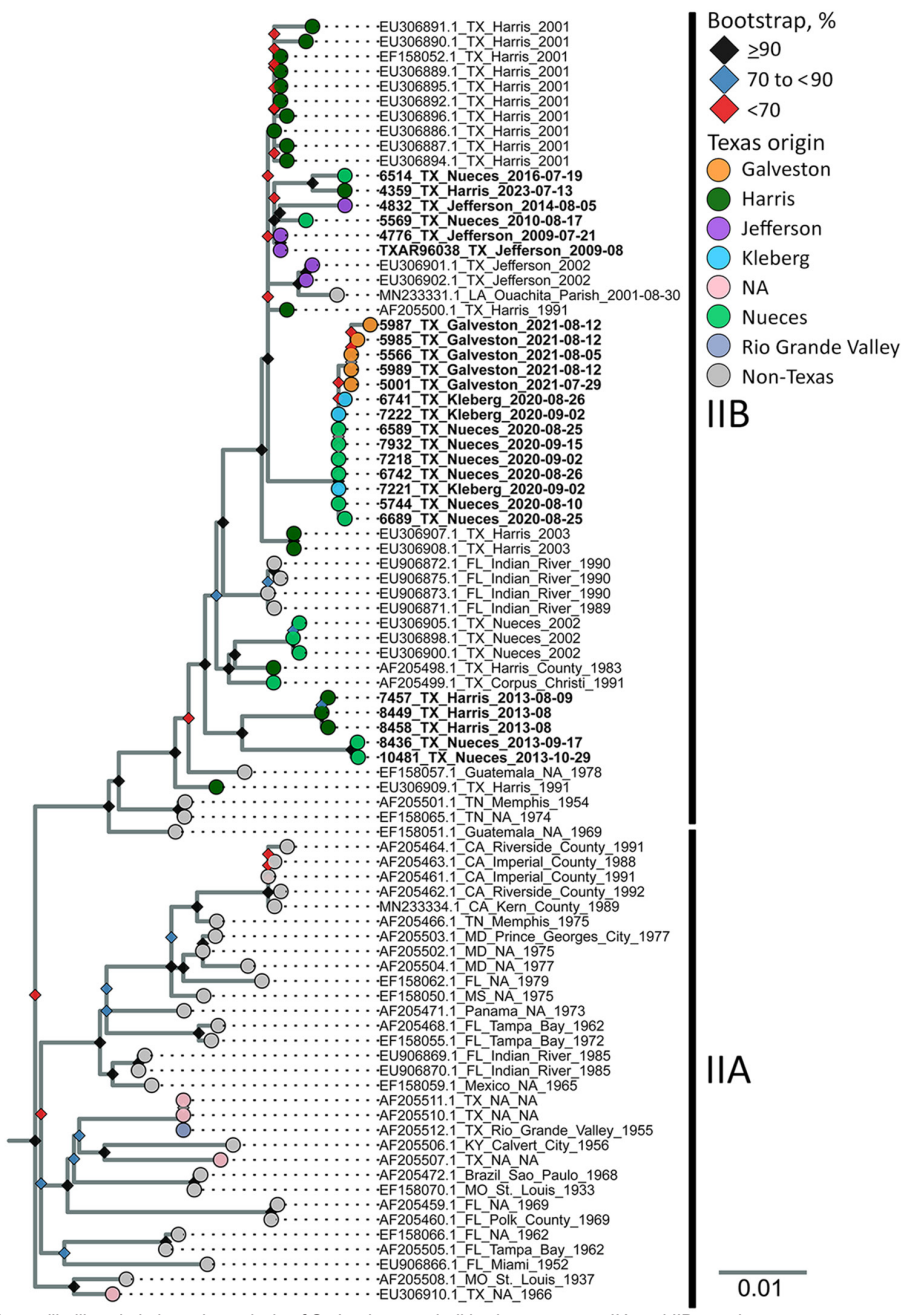
Maximum-likelihood phylogenic analysis of St. Louis encephalitis virus genotype IIA and IIB envelope gene sequences from study of circulation of genotype II and III St. Louis encephalitis virus, Texas, USA. Maximum-likelihood inferred tree of 86 SLEV genotype II envelope gene nucleotide sequences. Two genotype III outgroup sequences (not shown) were used to root tree (GenBank accession nos. FJ753287.2 and FJ753286.2). Tip labels indicate the GenBank accession number, sample origin, and collection date. Bold tip labels indicate samples generated by this study. Tip colors highlight different sample origins from within Texas, by county (or area, in the case of the Rio Grande Valley sample). Bootstrap support is shown at nodes; different colors indicate level of bootstrap support from 10,000 ultrafast bootstrap replicates. Scale bar indicates number of nucleotide substitutions per site. NA, not available (missing or unknown).

Because only a limited number of whole-genome sequences of genotype II SLEV were publicly available (n = 28), we used the envelope gene for phylogenetic analysis to increase the sample size spanning a broader timeframe (n = 61 publicly available sequences) (Figure 5). That analysis indicated that these samples fell within 2 clusters in genotype IIB. Our samples all share a basal node from a 1991 Harris County sample, but another more derived sample from 1983 indicates persistence in the region since at least the 1980s. Harris and Nueces County samples from 2013 (n = 5) clustered together in a position basal to the other samples from our dataset. Other samples from Galveston, Harris, Jefferson, Kleberg, and Nueces Counties (n = 20) span from 2009 to 2023 and clustered into a polytomy with other previously sequenced samples from Harris and Jefferson Counties, including a sample from Louisiana. This clade has a basal node of 2 samples from Harris County collected in 2003. Samples from Kleberg, Nueces, and Galveston Counties (n = 14) from 2020 and 2021 all clustered together, to the exclusion of the 6 remaining samples from Harris, Jefferson, and Nueces Counties that had collection dates spanning from 2009 to 2023. We compared the pairwise percentage identity of each genotype II sequence and determined that, across both genotype IIA and IIB, they differed in sequence identity by at most 4.5% (Appendix Figure 2) (*27*). Within genotypes IIA and IIB, we found a 2.7% difference in sequence identity in the envelope gene.

### Bayesian Phylogenetic Analysis of Genotype III Genomes

**Figure 6:**
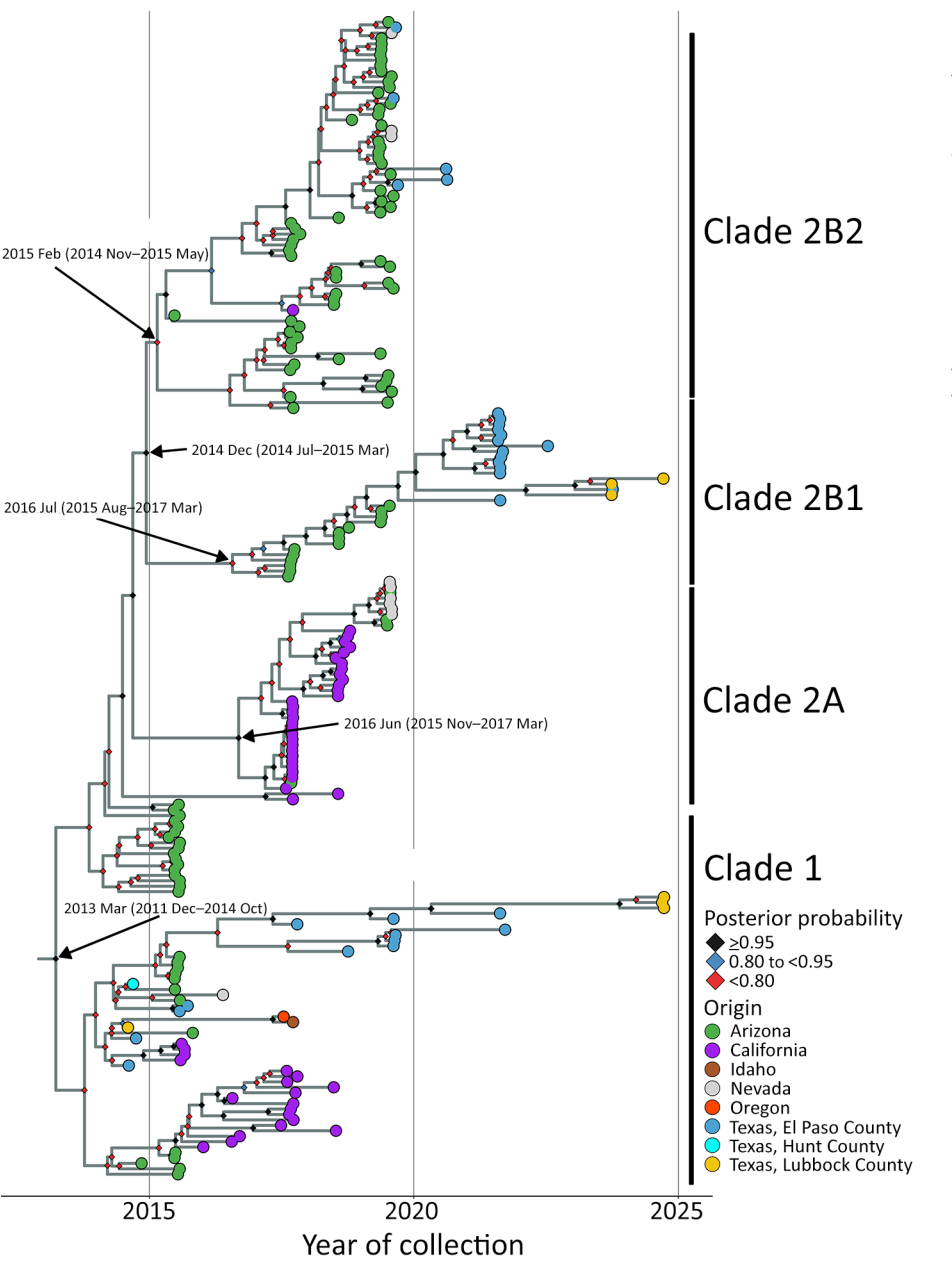
Bayesian phylogenetic analysis of SLEV genotype III from Texas and other states from study of circulation of genotype II and III St. Louis encephalitis virus, Texas, USA. Maximum clade credibility time tree of 216 whole genomes inferred by using BEAST (*34*). Tip colors reflect sample’s place of origin. Texas samples are further subdivided by county. Clade annotations are indicated to right of tips. Posterior probability for each node is indicated at node with a colored diamond. The *y*-axis scales in years are based on date of sample collection. Arrow labels indicate time to most recent common ancestor and 95% highest posterior density for each clade.

Given the epidemic potential of genotype III and to better understand introduction events and divergence times, we performed a more rigorous phylogenetic analysis by using Bayesian methods. TempEST (*33*) identified sufficient temporal signal (correlation coefficient 0.8284) in our genotype III dataset by using the root-to-tip distance compared with the date of collection from a maximum-likelihood tree inferred from the 216 genotype III genomes available (38 of our assemblies and 178 GenBank assemblies). The Bayesian time tree recapitulated a similar topology to a previous analysis (*21*), which showed US samples forming 2 clades (clades 1 and 2) (Figure 6); the node of the time to most recent common ancestor (tMRCA) of genotype III in the United States was March 2013 (95% highest posterior density [HPD] interval December 2011–October 2014).

Clade 1 primarily contained US samples collected during 2014–2024 and included the country’s earliest known genotype III sample, a SLEV- and WNV-positive *Cx. tarsalis* pool collected in July 2014 from Lubbock County. Clade 1 also contained 3 additional SLEV- and WNV-positive mosquito pools from El Paso County. Furthermore, this clade contained Lubbock County samples from 2024 with a well-supported basal node from a 2017 El Paso sample, suggesting sustained circulation of genotype III virus in West Texas. Other El Paso County samples from 2019 and 2021 did not form well-supported subclades, indicating possible introduction events from other regions. The newly sequenced 2014 Texas samples were not basal to the other US genotype III sequences, leaving the origin of genotype III introduction into the US unresolved.

Clade 2 consisted of samples from 2017 onward. The Bayesian time tree recapitulated subclades 2A, which were largely California samples (tMRCA June 2016 [95% HPD interval November 2015–March 2017]), and 2B (tMRCA December 2014 [95% HPD interval July 2014–March 2015]), which generally were Arizona samples. The Texas samples were within subclade 2B, clustering with the Arizona samples and subsequently forming 2 moderately supported subclades we called 2B1 (posterior probability 0.7898) and 2B2 (posterior probability 0.7933). Subclade 2B1 (tMRCA June 2016 [95% HPD interval August 2015–March 2017]) had limited sampling over multiple years from El Paso, Wichita, and Lubbock Counties, so determining persistence from year to year is limited. The Texas samples in this subclade were related to samples collected from Arizona, indicating an introduction from that region. The Texas samples bifurcated into sister clades; El Paso County samples clustered to the exclusion of Wichita and Lubbock Counties, although both clades shared a basal node from a 2021 El Paso County sample. In both groups, samples from >2 years are present (2021 and 2022 from El Paso County and 2023 and 2024 from Lubbock County), suggesting persistence of those viruses. Subclade 2B2 (tMRCA February 2015 [95% HPD interval November 2014–May 2015]) is a radiation with poorly supported virus population structure. This subclade is dominated by samples from Arizona; only 5 Texas samples (El Paso County 2019 and 2020) clustered into this group. These samples probably indicate introduction events into El Paso County from outside Texas that failed to persist or have remained undetected in subsequent years. El Paso County (2019 and 2021) and Lubbock County (2024) had clade 1 and clade 2 viruses, indicating SLEV genetic diversity within the same county. Collectively, the Bayesian phylogenetic analysis indicated repeated introduction events of genotype III into Texas and persistence of phylogenetically distinct viruses falling into clades 1 and 2.

## Discussion

This study highlights the importance of banking and sharing mosquito pool samples and collaborative surveillance efforts. Our retrospective study identified circulation of SLEV genotypes II and III in Texas during 2009–2024 and showed geographic segregation of each genotype, addressing a major gap in knowledge of genotype distribution in the state. Texas has 254 counties each with different mosquito sampling and pathogen testing algorithms, including some with no sampling or screening. Our sample size and scope were limited to those areas that tested and reported SLEV-positive mosquitoes. Of the 173 SLEV-positive mosquito pools reported since 2002 by Texas Department of Safety and Health Services, 43.3% (n = 75) were available, and 74 of those pools were collected after 2012. Because of availability of banked samples, our analysis did not include pools from the northern and western Texas counties before the introduction of genotype III, and it remains unknown which genotype or genotypes circulated immediately before the introduction. Further surveillance is needed to enhance the temporal resolution and improve classifications of introduction events from established virus lineages for SLEV and other arboviruses.

Genotypes I, II, and V SLEV historically are reported in Texas, and genotype II has dominated the Gulf Coast region since the 1980s (*16*,*35*). In line with previous findings, our results indicate that genotype IIB is still circulating >40 years after becoming dominant in the region. We detected different genotype IIB variants circulating in Nueces and Harris Counties at different times. The 2013 samples from Nueces and Harris Counties shared a recent common ancestor with samples from the same counties from 1991, 1983, and 2002, suggesting that the 2013 samples stem from an older lineage of genotype II that had not been sampled for at least a decade. No genotype III–positive samples were detected in the Gulf Coast region (Cameron, Galveston, Harris, Jefferson, Kleberg, and Nueces Counties) during 2009–2024 but were present during 2014–2024 in northern and western Texas counties (El Paso, Hunt, Lubbock, and Randall). These genotype III samples included the earliest known examples of genotype III virus in the United States. Our new data indicate that the genotype III introduction timeframe was December 2011–October 2014, differing slightly from previous reports of September 2011–May 2014 (*21*) and August 2012–October 2013 (*22*), suggesting that genotype III was circulating in the United States well before it was first detected in 2014. Our 2014 Texas samples were not basal to the other genotype III samples, indicating that the introduction site to the United States is still unknown. The earliest genotype III sample from Lubbock County was related to samples collected in Oregon and Idaho in 2017, indicating that Texas may have been an early area of genotype III introduction and that genotype III may have been exported to parts of the West Coast. We detected phylogenetically related viruses across years in clade 1 (early introduction) and clade 2 (later introduction), indicating establishment in Texas and greater viral diversity compared with Arizona and California, where clade 1 circulation is no longer reported (*21*,*22*). We detected introduction events with no direct ancestors from Texas in all clades, suggesting that West Texas is a site of continuous introduction events of SLEV genotype III.

The importation and circulation of new SLEV genotypes in West Texas, particularly in El Paso County, is not a new phenomenon. In the early 2000s, genotype VA circulated in El Paso County, whereas genotype IIB circulated in the Gulf Coast region (*35*). The El Paso isolates were genetically similar to genotype VA from South America that was contemporaneously circulating in California (*16*,*35*). Others hypothesized that cooler regions, such as El Paso County, require annual reintroduction of SLEV because overwintering does not appear to be effective for virus maintenance; however, in the subtropical Texas Gulf Coast region, SLEV can overwinter by unknown means (*35*). In contrast, our data supports year-to-year persistence of related genotype III viruses in El Paso County and potential SLEV overwintering in the county. Given the dispersal of SLEV occurs South-to-North from Central and South America to North America (*36*), and given the importance of the Texas Gulf Coast region in the Central Flyway for migratory birds from Central and South America (*37*), we would expect the Gulf Coast region rather than West Texas to be a major site for SLEV introductions. Further work is necessary to determine which migratory birds in West Texas are facilitating SLEV introduction events and how genotype III is overwintering in West Texas.

The observation that western and northern Texas counties harbor a different circulating genotype than Gulf Coast counties was previously reported and probably is likely driven by differences in rural–urban transmission cycles shaped by *Culex* mosquito distribution (*16*,*35*). *Cx. tarsalis* mosquitoes, typically involved in the rural transmission cycle of SLEV and associated with sporadic human infections (*38*,*39*), are better adapted to habitats in western Texas (*40*). *Cx. quinquefasciatus* mosquitoes, the primary vector in the urban transmission cycle and a driver of SLEV epidemics (*25*,*26*,*41*,*42*), are better adapted to habitats along the Gulf Coast region (*40*). Our samples generally align with this geographic dichotomy: all West Texas pools contained *Cx. tarsalis* mosquitoes, and 56% of Gulf Coast pools contained *Cx. quinquefasciatus* mosquitoes. SLEV genotypes and variants circulating in West Texas’ rural cycle can invade and establish urban cycles in more populated parts of Texas, resulting in SLEV outbreaks. Spillover from the SLEV rural cycle in West Texas to the Dallas metropolitan area in the 1960s probably resulted in outbreaks (*41*). Although we detected a SLEV genotype III–positive *Cx. quinquefasciatus* mosquito pool from 2014 in Hunt County, bordering the Dallas metropolitan area, this virus probably was an introduction from outside of Texas. Continued surveillance is necessary to determine whether genotype III spillover from West Texas into metropolitan areas is occurring, potentially initiating urban transmission cycles that could drive SLEV outbreaks.

Our data indicate that SLEV genotype III is established in Texas and importation events continue in the western counties. Despite genotype III circulation in Texas for nearly a decade, human cases have not increased. Since 2002, SLEV is a reportable disease in the state, where case counts are collected by the state contingent on diagnoses. The lack of cases in the northern and western counties could be attributable to several factors, such as the lack of clinical awareness to distinguish SLEV from WNV, the lack of incentive to test for arboviruses, potential misdiagnosis as WNV because of assay cross-reactivity, or the inability of SLEV genotype III from the rural transmission cycle to infect a large enough human population that it would be detected (*43*,*44*). Although genotype IIB remains the dominant strain in the Gulf Coast region >40 years after its introduction, whether genotype III possibly was introduced but failed to establish is unclear, given the limitations of sampling and testing in our retrospective study. Ongoing surveillance is critical to ensure that genotype III–associated outbreaks do not go undetected in affected counties and to monitor whether this genotype spreads to the Gulf Coast where an urban cycle with *Cx. quinquefasciatus* mosquitoes could cause epidemics. Genotype III became the dominant genotype in the western United States in <5 years, raising important questions about why a similar shift did not occur throughout Texas after nearly a decade. Whether any possible geographic and ecologic factors may be preventing the establishment of other genotypes in the Gulf Coast region is unknown. Incorporating accurate clinical data with robust mosquito surveillance data would provide a clearer measure of surveillance coverage across the state, which is currently unknown. Collectively, this study demonstrates the establishment and persistence of SLEV genotype III in Texas, which co-circulates with WNV and can cause concurrent outbreaks, underscoring the need for heightened clinical vigilance and ongoing surveillance.

Appendix Additional information about geographically distinct circulation of genotype II and III St. Louis encephalitis virus, Texas, USA, 2009–2024.
