# Supplementary material for: Geographically Distinct Circulation of Genotype II and III St. Louis Encephalitis Virus, Texas, USA, 2009–2024
Source: Emerg Infect Dis. 2026 Apr;32(4):521–32. doi: 10.3201/eid3204.250934 (PMC13094854; doi:10.3201/eid3204.250934)
Supplement: Appendix — Additional information about geographically distinct circulation of genotype II and III St. Louis encephalitis virus, Texas, USA, 2009–2024. [file 25-0934-Techapp-s1.pdf]

Article DOI: https://doi.org/10.3201/eid3204.250934

EID cannot ensure accessibility for supplementary materials supplied by authors. Readers who have difficulty accessing supplementary content should contact the authors for assistance.

Geographically Distinct Circulation of Genotype II and III St. Louis Encephalitis Virus, Texas, USA, 2009–2024

Appendix

Supplementary Methods

Mosquito Pools

Mosquito pools were provided by either the Texas Department of Safety and Health Services, Harris County Public Health Mosquito and Vector Control Division, or the Texas Tech University, Biological Threat Research Laboratory.

Texas Department of Safety and Health Services Mosquito Collection and Testing

The Texas Department of State Health Services Arbovirus/Entomology Laboratory received mosquito trap collections from local jurisdictions throughout the state for species identification and arbovirus testing. Mosquitoes were identified to species using standard taxonomic keys (*1*) and sorted by date of collection, trap location, and trap type. Female vector species were pooled into groups of 1-50 mosquitoes per tube and stored at -80°C until tested. Mosquito pools were homogenized in 1.5 ml of diluent (1% bovine albumin, 0.2 M Tris buffer, 8.76 g sodium chloride per liter, 1 ml phenol red [1.5%] aqueous solution per liter, 5 mg amphotericin B per liter, 0.05 g gentamicin sulfate per liter) with a single steel ball bearing using a Mixer Mill MM 400 (Retsch GmbH, Germany) at 25 cycles/s for 4 min. Homogenates were then centrifuged at 10,000 rpm for 5 min at 4°C. Cell culture screening was used for mosquito testing from 2009-2016 and real-time RT-PCR was used from 2017-2023. For cell culture screening, mosquito pool homogenates were inoculated into Vero and BHK cell cultures and monitored for cytopathic effects (CPE) for 10 days. If CPE was detected, then an immunofluorescence assay was performed for virus identification. For real-time RT-PCR testing,

RNA was extracted from 50 µl of the mosquito pool homogenate supernatant using a MagMAX-96 Viral RNA Isolation Kit (Thermo Fisher Scientific, MA) with the KingFisher Flex Magnetic Particle Processor (Thermo Fisher Scientific, MA) according to the manufacturer's protocol. The extracted RNA was tested using a real-time RT-PCR assay for the detection of WNV, SLEV, EEEV, and WEEV (*2*,*3*).

Harris County Public Health Mosquito and Vector Control Division Mosquito Collection and Testing

The Harris County Public Health Mosquito and Vector Control Division sets and retrieves mosquito traps with dedicated surveillance staff from each of the 268 Mosquito and Vector Control operational areas (MVCOAs) encompassing the entire county. Each MVCOA consists of either a modified Centers for Disease Control and Prevention (CDC) gravid (GV) trap or a modified CDC miniature light trap, while specific areas containing an additional Biogents Sentinel (BG) trap for surveillance. Trap collections were assigned collection numbers and organized by MVCOA, trap collection date, and trap type. Mosquito subsamples from collections were identified to species using standard identification keys and guides (*1*,*4*). Female vectors of public health significance were sorted on chill tables and pooled into 1.5 mL microcentrifuge tubes consisting of 1-50 mosquitoes of the same species. All microcentrifuge tubes were stored at -80°C prior to testing. Each mosquito pool was homogenized in 1.7 mL of BA-1 diluent consisting of 100 mL of 10× M-199 medium with Hank's salts per liter, supplemented with 0.05 M Tris buffer (pH 7.6), 1% bovine serum albumin, 0.34 g/L sodium bicarbonate, 1 mL/L amphotericin B (250 mg/mL), 1 mL/L gentamicin sulfate (50 mg/mL), and 1 mL/L penicillin-streptomycin (10,000 U penicillin and 10 mg/mL streptomycin). Homogenization was performed with a single copper-coated steel ball bearing using a Qiagen TissueLyser II at 25 Hz for 12 minutes, followed by centrifugation at 10,000 rpm for 12 minutes at 4°C.

From 2009 to 2023, a modified CDC Enzyme-Linked Immunosorbent Assay (ELISA) was used to detect St. Louis encephalitis virus (SLEV) antigen in mosquito homogenates (*5*). The assay employed a virus-specific capture monoclonal antibody (4A4C-4, IgG) and a horseradish peroxidase (HRP)-conjugated detecting antibody (6B6C-1). Plates were coated with capture antibody, incubated (2–24 hours), washed, and blocked with PBS containing 1% BSA

and incubated for one hour at 37°C. After removal of the blocking solution, mosquito pools and controls were added and incubated overnight at 4°C. Plates were washed, then treated with the detecting antibody for one hour at 37°C. Substrate solution (hydrogen peroxide and TMB) was added, and color development was stopped with sulfuric acid.

Optical density (OD) was measured using a Biotek spectrophotometer. Samples were considered positive if their OD was ≥2× the mean OD of the negative controls. Positive pools were submitted to UTMB, CDC-DVBID (Fort Collins, CO), or the Texas Department of State Health Services (DSHS) for confirmation.

Texas Tech University Biological Threat Research Laboratory Mosquito Collection and Testing

Texas Tech University Biological Threat Research Laboratory samples were either collected by laboratory individuals or City of Lubbock Vector Control technicians. Samples were collected using encephalitis vector survey traps, identified to species using standard taxonomic keys (*1*) and sorted by date of collection, trap location, and trap type. Female *Culex quinquefasciatus, Culex tarsalis,* and *Aedes vexans* were pooled (1-35 individuals) by species into a tube containing two sterilized steel ball bearings and stored for no more than one week at -20°C. Each pool was homogenized first by adding 1X sodium chloride-tris-EDTA and proteinase K mixture to the tube, placing the tube on a VWR Bead Mill Homogenizer, and homogenizing at speed 4 for 60 seconds. Mosquito lysate homogenates were centrifuged at 17,000g for 10 min. Mosquitoes were tested immediately following centrifugation or stored for one year at -20°C. For real-time RT-PCR testing, RNA was extracted from 140 µl of the mosquito pool homogenate supernatant using QIAamp® Viral RNA Mini Kit. Samples were extracted manually or using a QIACube® according to the manufacturer's protocol. The extracted RNA was tested using a real-time RT-PCR assay for the detection of WNV, SLEV, EEEV, and WEEV (*2*,*3*).

Mosquito pool metadata can be found in Appendix Table 1 (*6*). Since virus screening modalities varied based on where the pools originated, all pools were screened again by Baylor College of Medicine using the RT-PCR assay mentioned below to confirm they were virus positive.

Viral Isolation

Viral isolation was performed by incubating mosquito pool homogenate with Vero CCL81 cells. In short, mosquito pool homogenates were thawed on ice and 50 uL of homogenate

was mixed with 150 uL of DMEM and 200 uL of DNA/RNA Shield reagent and stored at -20°C until RNA extraction. Another 50 uL of homogenate was mixed with 5 mL of DMEM containing 2%FBS and 1x anti-mycotic/anti-biotic solution and was put on Vero CCL81 cells that were ~80% confluent in a T-75 flask. After one hour of incubation at 37°C, 5% CO2, another 15mL of DMEM containing 2% FBS and 1x anti-mycotic/anti-biotic solution was added to the flask. Infected flasks were incubated at 37C, 5% CO2 until frank cytopathic effects were visible compared to uninfected control flasks (usually 3-7 days after start of infection). When cytopathic effects were apparent, we centrifuged the cell culture supernatant to remove cell debris (500g for 5min). Cell culture supernatant was aliquoted and stored at -80°C. We also mixed 50 uL of centrifuged cell culture supernatant with 150 uL of DMEM and 200 uL of 2x DNA/RNA Shield reagent and stored at -20°C until RNA extraction. For the four Texas SLEV isolates (V07457, V08449, V08458, and TX AR 9-6038) that were available from the Biodefense and Emerging Infections Research resources repository (BEI), we thawed stock tubes from BEI on ice and mixed 50 uL of stock with 150 uL of DMEM and 200 uL of 2x DNA/RNA Shield reagent and stored at -20°C until RNA extraction.

RNA Isolation and SLEV RT-PCR Testing

Total RNA and DNA were isolated from samples using a modified Zymo Quick-DNA/RNA Pathogen Miniprep protocol. Notably, we added 16 uL of proteinase K (20 mg/mL) to the inactivated homogenate or BEI stock virus in DNA/RNA Shield reagent and incubated this at 56°C for 15 min prior to adding the Pathogen DNA/RNA Buffer. The Zymo-Spin IIICG column was used in place of the Zymo-Spin IICR column. No DNase-I treatment was performed on the samples. An extraction negative control was performed with every batch of extraction whichconsisted of nuclease-free water in place of homogenate. Extracted nucleic acid and remaining homogenate were stored at -80C. We tested all samples for the presence of SLEV RNA using a modified protocol from Lanciotti et al. (*7*). Briefly, we used the qScript XLT 1-Step RT-qPCR ToughMix, Low ROX mastermix on a ThermoFisher QuantStudio 3 instrument. We used RNA from the TX AR 9-6038 BEI stock virus as a positive control for each plate run. We formulated the mastermix conditions following the manufacturer's recommendation using 600nM/500nM SLE2420 and SLE2487c primers, respectively, with 300nM of FAM-SLE2444-ZEN- IABkFQ probe. Cycling conditions were as follows: Hold stage [50°C for 10 min, 95°C for 1 min] 1x, PCR stage [95°C for 5s, 60°C for 45s] 45x.

Tiled-Amplicon Sequencing Primer Scheme Development

We designed a tiled-amplicon primer scheme to amplify the entire coding sequence of the SLEV genome using the Olivar tool (v1.1.4) (*8*). SLEV genomes with complete coding sequences were obtained from GenBank and genomes with ≥30% ambiguous bases were removed using a custom python script (ambiguity_filter.py) (*6*). The remaining genomes were clustered with CD-HIT-EST (v4.8.1) (*9*) using a sequence identity threshold of 99%. We genotyped a representative from each cluster using a phylogenetic analysis. The nucleotide sequences were aligned using MAFFT (v7.490) (*10*) with the --auto setting. We inferred a maximum-likelihood tree using IQ-TREE2 (*11–13*) and visualized this in ITOL (v7) (*14*). We selected at most five genome assemblies from each genotype, excluding the Palenque isolates, which are only ~82% similar to the other genotypes. The selected genome assemblies (listed in (*6*)) were aligned with MAFFT using the --auto setting. The per-base frequency of each position was determined relative to the SLEV reference genome (DQ525916.1) using msa_base_frequency.py (*6*). The off-target database was generated with mosquito, human, and African green monkey genomes (*6*) using NCBI+ (v2.16.0+) (*15*). Olivar was given the reference genome (DQ525916.1), the per-base frequency, and the off-target database to generate a primer scheme with max amplicon length of 1.4kb. We manually inspected the primer scheme to ensure complete coverage of the coding sequence and sufficient overlap between amplicons. Primer sequences were individually inspected to verify that they did not fall in polymorphic areas (particularly the 3' end of the primers). Primers that did (4rP, 5fP, 7rP, 9Fp, 9rP, 10fP) were manually adjusted to avoid these areas even if these increased the size of the amplicon slightly beyond 1.4kb. Manually adjusted primer pools were validated using the validate function of Olivar to ensure that there were no off-target amplicons that would be generated or issues with other primers in the multiplex pool (*6*). The final primer scheme can be found in (*6*). Primers were ordered from Sigma resuspended to 100 uM in water. Pools A and B were made by mixing 10uL from each primer to make a 5x stock. We made working stocks by diluting the 5x stock to 1x with nuclease-free water.

Viral Genome Amplification and Sequencing

Using our Olivar-designed primer scheme, we generated SLEV tiled amplicons via PCR. We mixed 7uL of nucleic acid from each sample with 1 uL of random primers and 2 uL of 5x qScript Ultra reaction mix and incubated following manufacturer's instructions. Two different

PCRs using either primer pool A or B were performed following cDNA synthesis with 2.5 uL of cDNA mixed with 1.8 uL of primer pool, and 6.25 uL of Q5 2x mastermix. Cycling conditions were as follows: 98°C, 30s, 1x; 98°C, 15s, 61°C 2min, 65°C 3 min, 35x; 4°C hold. PCRs were stored at 4°C until the following day when pool A and pool B PCRs were pooled for each sample and quantified using a Qubit 4 with the 1x dsDNA Broad Range kit. We normalized the pooled samples to 12 ng/uL in 10 uL of nuclease free water. Each sample was barcoded using the SQK-RBK114.96 kit by mixing 5 uL of normalized sample with 2.5 uL of nuclease free water and 2.5 uL of rapid barcode and incubating for 2 mins at 30°C and 2 min at 80°C. The library was sequenced on an R10.4.1 flow cell either on a PromethION P2 Solo or MinION Mk1B instrument. Sequencing data was acquired using MinKnow (v24.11.10) and basecalled with dorado (v0.9.1) using the SUP model (v5.0.0).

Consensus Genome Assembly

Consensus viral genome assemblies were generated using the ViralRecon nextflow pipeline (*16*), using the ARTIC minion pipeline, with modifications. We made modifications to modules_nanopore.config to increase read length filtering to be between 200bp and 1,500bp by default, and a custom config file for our primer scheme (files found here (*6*)). Consensus genomes with >30% ambiguous bases were excluded from phylogenetic analyses (ambiguity_filter.py). Genomes assembled from viral isolations were excluded from phylogenetic analysis when genomes from the originating mosquito pools were more complete.

Genome completeness vs Ct

We calculated the completeness of our viral assemblies by using the completeness_calculation.py script. Briefly, the completeness_calculation.py script determines the percentage of non-ambiguous bases present in the assemblies while accounting for the 323 bases of the non-coding portion of the SLEV reference genome (DQ525916.1) not covered by our primer scheme, see equation below:

$$\%\,completeness = \left(\frac{10{,}617\ bases - \ (\#\ of\ ambiguous\ bases - 323\ bases)}{10{,}617\ bases}\right) *\ 100\%$$

Completeness was calculated for each sample and plotted against that sample's Ct value from the RT-PCR previously mentioned. We plotted the data using ggplot2 in R (*6*). We plotted five representative samples that spanned different Ct values and genome completeness to

visualize read distribution across the SLEV genome. Primer trimmed BAM files generated from the ViralRecon pipeline were used in the genome_coverage_script.py to produce Appendix Figure 2.

Maximum-likelihood Analysis of SLEV Genotypes and SLEV Genotype II Envelope

To determine the phylogenetic placement of our SLEV assemblies, we inferred a maximum-likelihood tree of all SLEV genotypes with available genomes on GenBank. SLEV genomes were obtained from GenBank and filtered to remove genomes with >30% ambiguous bases using ambiguity_filter.py. The nucleotide sequences were aligned using MAFFT (v7.490) with the --auto setting (*10*). We inferred a maximum-likelihood tree using IQ-TREE2 with 10,000 ultrafast bootstrap replicates (*11*,*12*). The best-fit model as determined by ModelFinder (*17*) was GTR+F+G4.The same process was used for SLEV genotype II envelope sequences. The best-fit model for the envelope tree as determined by ModelFinder was TIM2e+G4. Visualization and annotation of the phylogenies was done in R using ggtree (*18*) and InkScape. Newick tree files for each phylogeny are available (*6*).

Bayesian Phylogenetic Analysis of SLEV Genotype III

We assessed the maximum-likelihood tree of all SLEV genotype III genomes with root-to-tip genetic divergence and time of sampling regression using TempEst (*19*). We determined from this analysis that temporal signal warranted generating a timetree (correlation coefficient = 0.8284). Bayesian phylogenetic analysis used BEAST (v1.10.4) (*20*). We used an uncorrelated relaxed clock model, a GTR+F+G4 substitution model, and a Bayesian skyline with a 2 groups tree prior with MCMC chains of length 100, ,000,000 and sampling every 10,000 trees to infer the timetree. Two independent MCMC chains were generated, and convergence was confirmed in each chain with Tracer prior to combining with LogCombiner with 10% burn-in for each log and tree file. The maximum clade credibility tree was generated with TreeAnnotator. The XML generated by BEAUti for running BEAST and the nexus file generated by TreeAnnotator can be found at (*6*). Phylogeny visualization and annotation were done using ggtree in R and Inkscape.

Appendix Table 1. Mosquito pool metadata

Pool#	Virus	CT Value	Date of Collection	County	Species	Provider	Virus Isolated	Genome Completeness	Sample Used for Analysis	SLEV Genotype	Sequencing Platform
3097	SLEV	29.2	2022-07-07	El Paso	*Cx. tarsalis*	TX DSHS	YES	50.16	NO	III	MinION Mk1B
4776	SLEV	25.2	2009-07-21	Jefferson	*Cx. quinquefasciatus*	TX DSHS	NO	100.00	YES	II	MinION Mk1B
4792	SLEV	25.3	2020-07-30	El Paso	*Cx. tarsalis*	TX DSHS	NO	0.00	NO	III	n/a
4799	SLEV	21.2	2021-07-27	El Paso	*Cx. tarsalis*	TX DSHS	NO	55.06	YES (Genotyping only)	III	PromethION P2S
4832	SLEV	22.7	2014-08-05	Jefferson	*Cx. quinquefasciatus*	TX DSHS	NO	100.00	YES	II	MinION Mk1B
4992	SLEV	28.9	2014-08-05	El Paso	*Cx. quinquefasciatus*	TX DSHS	YES	0.00	NO	III	n/a
5001	SLEV	19.9	2021-07-29	Galveston	*Cx. quinquefasciatus*	TX DSHS	YES	99.99	YES	II	PromethION P2S
5016	SLEV	21.9	2021-07-29	El Paso	*Cx. quinquefasciatus*	TX DSHS	YES	94.99	NO	III	MinION Mk1B
5017	SLEV	16.2	2021-07-29	El Paso	*Cx. tarsalis*	TX DSHS	YES	99.99	YES	III	MinION Mk1B
5019	SLEV	17.6	2021-07-29	El Paso	*Cx. tarsalis*	TX DSHS	YES	100.00	YES	III	MinION Mk1B
5193	SLEV	20.9	2020-08-04	El Paso	*Cx. quinquefasciatus*	TX DSHS	YES	90.89	NO	III	PromethION P2S
5292	SLEV	22.16	2021-08-03	El Paso	*Cx. quinquefasciatus*	TX DSHS	YES	99.88	YES	III	PromethION P2S
5538	SLEV	21.9	2021-08-05	El Paso	*Cx. tarsalis*	TX DSHS	YES	100.00	YES	III	PromethION P2S
5566	SLEV	27.9	2021-08-05	Galveston	*Cx. quinquefasciatus*	TX DSHS	YES	24.74	NO	II	MinION Mk1B
5569	SLEV	28.1	2010-08-17	Nueces	*Cx. quinquefasciatus*	TX DSHS	YES	99.98	NO	II	MinION Mk1B
5702	SLEV	31	2023-08-22	Randall	*Cx. tarsalis*	TX DSHS	NO	0.00	NO	n/a	n/a
5713	SLEV	24.3	2023-08-22	Randall	*Cx. tarsalis*	TX DSHS	NO	0.00	NO	n/a	n/a
5744	SLEV	28.6	2020-08-10	Nueces	*Cx. quinquefasciatus*	TX DSHS	YES	58.15	NO	II	MinION Mk1B
5754	SLEV	24.2	2020-08-11	El Paso	*Cx. quinquefasciatus*	TX DSHS	NO	69.71	YES	III	PromethION P2S
5783	WNV/ SLEV	22.47	2019-07-30	El Paso	*Cx. tarsalis*	TX DSHS	NO	0.00	NO	n/a	n/a
5985	SLEV	24.2	2021-08-12	Galveston	*Cx. quinquefasciatus*	TX DSHS	YES	100.00	YES	II	PromethION P2S
5987	SLEV	21.7	2021-08-12	Galveston	*Cx. quinquefasciatus*	TX DSHS	YES	100.00	YES	II	PromethION P2S
5989	SLEV	19.4	2021-08-12	Galveston	*Cx. quinquefasciatus*	TX DSHS	YES	99.99	YES	II	PromethION P2S
6023	SLEV	28.8	2022-09-07	El Paso	*Cx. tarsalis*	TX DSHS	NO	0.00	NO	n/a	n/a
6027	SLEV	21	2021-08-11	El Paso	*Cx. tarsalis*	TX DSHS	YES	100.00	YES	III	PromethION P2S
6029	SLEV	23.5	2021-08-12	El Paso	*Cx. tarsalis*	TX DSHS	NO	67.51	YES (Genotyping only)	III	PromethION P2S
6033	SLEV	18.5	2021-08-12	El Paso	*Cx. quinquefasciatus*	TX DSHS	YES	99.98	YES	III	MinION Mk1B
6034	SLEV	18.3	2021-08-12	El Paso	*Cx. tarsalis*	TX DSHS	YES	99.99	YES	III	PromethION P2S
6037	SLEV	27.4	2021-08-11	El Paso	*Cx. quinquefasciatus*	TX DSHS	NO	0.00	NO	n/a	n/a
6038	SLEV	17.7	2021-08-12	El Paso	*Cx. quinquefasciatus*	TX DSHS	YES	100.00	YES	III	MinION Mk1B
6048	SLEV	23.6	2021-08-11	El Paso	*Cx. tarsalis*	TX DSHS	YES	84.98	NO	III	MinION Mk1B
6210	SLEV	22.5	2019-08-06	El Paso	*Cx. tarsalis*	TX DSHS	YES	96.01	NO	III	PromethION P2S
6222	WNV/ SLEV	23.27	2019-08-06	El Paso	*Cx. tarsalis*	TX DSHS	YES	99.97	YES	III	MinION Mk1B
6224	SLEV	21.9	2019-08-06	El Paso	*Cx. tarsalis*	TX DSHS	YES	100.00	YES	III	PromethION P2S
6354	SLEV	32.8	2020-08-19	Cameron	*Cx. quinquefasciatus*	TX DSHS	NO	9.22	YES (Genotyping only)	II	MinION Mk1B
6411	SLEV	22.2	2021-08-19	El Paso	*Cx. quinquefasciatus*	TX DSHS	YES	86.22	NO	III	PromethION P2S
6412	SLEV	25.9	2021-08-19	El Paso	*Cx. tarsalis*	TX DSHS	YES	100.00	YES	III	MinION Mk1B
6514	SLEV	29.5	2016-07-19	Nueces	*Cx. quinquefasciatus*	TX DSHS	YES	75.31	NO	II	PromethION P2S
6589	SLEV	23.5	2020-08-25	Nueces	*Cx. quinquefasciatus*	TX DSHS	YES	99.95	YES	II	MinION Mk1B
6674	WNV/ SLEV	26.48	2019-08-13	El Paso	*Cx. tarsalis*	TX DSHS	YES	0.00	NO	III	n/a
6689	SLEV	20.6	2020-08-25	Nueces	*Cx. quinquefasciatus*	TX DSHS	YES	99.99	YES	II	PromethION P2S

Pool#	Virus	CT Value	Date of Collection	County	Species	Provider	Virus Isolated	Genome Completeness	Sample Used for Analysis	SLEV Genotype	Sequencing Platform
6741	SLEV	26.2	2020-08-26	Kleberg	*Cx. quinquefasciatus*	TX DSHS	YES	100.00	YES	II	MinION Mk1B
6742	SLEV	27.8	2020-08-26	Kleberg	*Cx. quinquefasciatus*	TX DSHS	NO	99.96	YES	II	MinION Mk1B
6822	SLEV	22.7	2023-09-13	Randall	*Cx. quinquefasciatus*	TX DSHS	NO	8.70	YES (Genotyping only)	III	PromethION P2S
6826	SLEV	26.7	2023-09-13	Randall	*Cx. tarsalis*	TX DSHS	NO	0.00	NO	n/a	n/a
7000	SLEV	24.1	2014-09-03	Hunt	*Cx. quinquefasciatus*	TX DSHS	YES	90.89	NO	III	MinION Mk1B
7153	WNV/ SLEV	21.55	2019-08-20	El Paso	*Cx. tarsalis*	TX DSHS	YES	100.00	YES	III	MinION Mk1B
7204	SLEV	20.4	2021-08-31	El Paso	*Cx. quinquefasciatus*	TX DSHS	YES	97.63	NO	III	PromethION P2S
7218	SLEV	23.9	2020-09-02	Nueces	*Cx. quinquefasciatus*	TX DSHS	NO	100.00	YES	II	MinION Mk1B
7221	SLEV	27.5	2020-09-02	Kleberg	*Cx. quinquefasciatus*	TX DSHS	YES	100.00	NO	II	MinION Mk1B
7222	SLEV	25.9	2020-09-02	Kleberg	*Cx. quinquefasciatus*	TX DSHS	YES	98.00	NO	II	PromethION P2S
7379	SLEV	27.2	2023-09-27	Wichita	*Cx. quinquefasciatus*	TX DSHS	NO	76.28	YES	III	PromethION P2S
7446	SLEV	20.5	2019-08-22	El Paso	*Cx. quinquefasciatus*	TX DSHS	YES	94.11	NO	III	MinION Mk1B
7932	SLEV	26.1	2020-09-15	Nueces	*Cx. quinquefasciatus*	TX DSHS	YES	90.16	NO	II	MinION Mk1B
8026	SLEV	22.6	2021-09-15	El Paso	*Cx. tarsalis*	TX DSHS	YES	100.00	YES	III	PromethION P2S
8323	SLEV	19.8	2019-09-05	El Paso	*Cx. tarsalis*	TX DSHS	YES	99.99	YES	III	MinION Mk1B
8415	SLEV	21.2	2014-09-24	El Paso	*Cx. tarsalis*	TX DSHS	YES	100.00	YES	III	MinION Mk1B
8436	SLEV	25.8	2013-09-17	Nueces	*Cx. quinquefasciatus*	TX DSHS	YES	85.10	NO	II	MinION Mk1B
8683	SLEV	26.3	2023-10-19	Wichita	*Cx. quinquefasciatus*	TX DSHS	NO	0.00	NO	n/a	n/a
10157	SLEV	19.3	2015-09-15	El Paso	*Cx. tarsalis*	TX DSHS	YES	99.97	YES	III	PromethION P2S
10328	SLEV	24.7	2018-09-27	El Paso	*Cx. tarsalis*	TX DSHS	YES	81.87	NO	III	MinION Mk1B
10481	SLEV	23.3	2013-10-29	Nueces	*Cx. quinquefasciatus*	TX DSHS	YES	85.09	NO	II	MinION Mk1B
11229	SLEV	21.6	2017-10-11	El Paso	*Cx. tarsalis*	TX DSHS	YES	95.25	NO	III	PromethION P2S
140473	SLEV/ WNV	23.68	2014-07-30	Lubbock	*Cx. tarsalis*	Lubbock	n/a	99.78	YES	III	MinION Mk1B
190164	SLEV	22.27	2019-09-24	Lubbock	*Cx. tarsalis*	Lubbock	n/a	0.00	NO	n/a	PromethION P2S
2300445	SLEV	22.2	2023-09-20	Lubbock	*Cx. tarsalis*	Lubbock	n/a	0.00	NO	n/a	PromethION P2S
2300447	SLEV	24.38	2023-09-20	Lubbock	*Cx. tarsalis*	Lubbock	n/a	72.83	YES	III	PromethION P2S
2400375	SLEV	26.79	2024-09-09	Lubbock	*Cx. quinquefasciatus*	Lubbock	n/a	24.83	YES (Genotyping only)	III	PromethION P2S
2400389	SLEV	17.18	2024-09-03	Lubbock	*Cx. tarsalis*	Lubbock	n/a	100.00	YES	III	PromethION P2S
2400405	SLEV	20.56	2024-09-16	Lubbock	*Cx. tarsalis*	Lubbock	n/a	99.89	YES	III	PromethION P2S
2400422	SLEV	23.6	2024-09-12	Lubbock	*Cx. tarsalis*	Lubbock	n/a	83.59	YES	III	PromethION P2S
2400431	SLEV	27.75	2024-09-16	Lubbock	*Cx. quinquefasciatus*	Lubbock	n/a	75.59	YES	III	PromethION P2S
2400450	SLEV	25.54	2024-09-24	Lubbock	*Cx. tarsalis*	Lubbock	n/a	11.49	YES (Genotyping only)	III	PromethION P2S
2300453-1	SLEV	24.91	2023-09-20	Lubbock	*Cx. tarsalis*	Lubbock	n/a	85.21	YES	III	PromethION P2S
4359	SLEV	28	2023-07-13	Harris	*Cx. quinquefasciatus*	MVCD	NO	100.00	YES	II	MinION Mk1B

Appendix Table 2. Virus Isolate Metadata

Isolate	Virus	CT Value	Date of Collection	County	Species	Provider	Virus	Sample Used for Analysis	Genome Completeness	SLEV Genotype	Sequencing Platform
3097	SLEV	14.6	2022-07-07	El Paso	*Cx. tarsalis*	TX DSHS	SLEV	YES	100.00	III	PromethION P2S
4992	SLEV	18.4	2014-08-05	El Paso	*Cx. quinquefasciatus*	TX DSHS	SLEV	YES	100.00	III	MinION Mk1B
5001	SLEV	11.3	2021-07-29	Galveston	*Cx. quinquefasciatus*	TX DSHS	SLEV	NO	100.00	II	PromethION P2S
5016	SLEV	11.73	2021-07-29	El Paso	*Cx. quinquefasciatus*	TX DSHS	SLEV	YES	100.00	III	PromethION P2S
5017	SLEV	12.8	2021-07-29	El Paso	*Cx. tarsalis*	TX DSHS	SLEV	NO	100.00	III	PromethION P2S
5019	SLEV	14.4	2021-07-29	El Paso	*Cx. tarsalis*	TX DSHS	SLEV	NO	100.00	III	PromethION P2S
5193	SLEV	8.7	2020-08-04	El Paso	*Cx. quinquefasciatus*	TX DSHS	SLEV	YES	100.00	III	MinION Mk1B
5292	SLEV	8.6	2021-08-03	El Paso	*Cx. quinquefasciatus*	TX DSHS	SLEV	NO	99.99	III	PromethION P2S
5538	SLEV	9.2	2021-08-05	El Paso	*Cx. tarsalis*	TX DSHS	SLEV	NO	100.00	III	PromethION P2S
5566	SLEV	16.9	2021-08-05	Galveston	*Cx. quinquefasciatus*	TX DSHS	SLEV	YES	99.98	II	PromethION P2S
5569	SLEV	12.3	2010-08-17	Nueces	*Cx. quinquefasciatus*	TX DSHS	SLEV	YES	99.98	II	PromethION P2S
5744	SLEV	13.8	2020-08-10	Nueces	*Cx. quinquefasciatus*	TX DSHS	SLEV	YES	100.00	II	PromethION P2S
5985	SLEV	12.8	2021-08-12	Galveston	*Cx. quinquefasciatus*	TX DSHS	SLEV	NO	100.00	II	PromethION P2S
5987	SLEV	12.5	2021-08-12	Galveston	*Cx. quinquefasciatus*	TX DSHS	SLEV	NO	100.00	II	PromethION P2S
5989	SLEV	10.4	2021-08-12	Galveston	*Cx. quinquefasciatus*	TX DSHS	SLEV	NO	99.99	II	PromethION P2S
6027	SLEV	8.4	2021-08-11	El Paso	*Cx. tarsalis*	TX DSHS	SLEV	NO	100.00	III	PromethION P2S
6033	SLEV	10.6	2021-08-12	El Paso	*Cx. quinquefasciatus*	TX DSHS	SLEV	NO	99.99	III	PromethION P2S
6034	SLEV	8.6	2021-08-12	El Paso	*Cx. tarsalis*	TX DSHS	SLEV	NO	99.98	III	PromethION P2S
6038	SLEV	8.9	2021-08-12	El Paso	*Cx. quinquefasciatus*	TX DSHS	SLEV	NO	99.99	III	PromethION P2S
6048	SLEV	9.6	2021-08-11	El Paso	*Cx. tarsalis*	TX DSHS	SLEV	YES	100.00	III	PromethION P2S
6210	SLEV	11	2019-08-06	El Paso	*Cx. tarsalis*	TX DSHS	SLEV	YES	100.00	III	PromethION P2S
6224	SLEV	9	2019-08-06	El Paso	*Cx. tarsalis*	TX DSHS	SLEV	NO	99.97	III	PromethION P2S
6411	SLEV	10.3	2021-08-19	El Paso	*Cx. quinquefasciatus*	TX DSHS	SLEV	YES	100.00	III	PromethION P2S
6412	SLEV	12.7	2021-08-19	El Paso	*Cx. tarsalis*	TX DSHS	SLEV	NO	100.00	III	PromethION P2S
6514	SLEV	15.3	2016-07-19	Nueces	*Cx. quinquefasciatus*	TX DSHS	SLEV	YES	99.97	II	PromethION P2S
6589	SLEV	10.3	2020-08-25	Nueces	*Cx. quinquefasciatus*	TX DSHS	SLEV	NO	100.00	II	PromethION P2S
6689	SLEV	11.2	2020-08-25	Nueces	*Cx. quinquefasciatus*	TX DSHS	SLEV	NO	99.99	II	PromethION P2S
6741	SLEV	12.3	2020-08-26	Kleberg	*Cx. quinquefasciatus*	TX DSHS	SLEV	NO	100.00	II	PromethION P2S
7000	SLEV	10.3	2014-09-03	Hunt	*Cx. quinquefasciatus*	TX DSHS	SLEV	YES	100.00	III	PromethION P2S
7204	SLEV	9	2021-08-31	El Paso	*Cx. quinquefasciatus*	TX DSHS	SLEV	YES	99.99	III	PromethION P2S
7221	SLEV	16.6	2020-09-02	Kleberg	*Cx. quinquefasciatus*	TX DSHS	SLEV	YES	100.00	II	PromethION P2S
7222	SLEV	12.1	2020-09-02	Kleberg	*Cx. quinquefasciatus*	TX DSHS	SLEV	YES	100.00	II	PromethION P2S
7446	SLEV	7.7	2019-08-22	El Paso	*Cx. quinquefasciatus*	TX DSHS	SLEV	YES	100.00	III	MinION Mk1B
7932	SLEV	17.8	2020-09-15	Nueces	*Cx. quinquefasciatus*	TX DSHS	SLEV	YES	100.00	II	PromethION P2S
8026	SLEV	9.7	2021-09-15	El Paso	*Cx. tarsalis*	TX DSHS	SLEV	NO	99.88	III	PromethION P2S
8323	SLEV	9.4	2019-09-05	El Paso	*Cx. tarsalis*	TX DSHS	SLEV	NO	99.98	III	PromethION P2S
8415	SLEV	8.7	2014-09-24	El Paso	*Cx. tarsalis*	TX DSHS	SLEV	NO	100.00	III	PromethION P2S
8436	SLEV	15.3	2013-09-17	Nueces	*Cx. quinquefasciatus*	TX DSHS	SLEV	YES	100.00	II	PromethION P2S
10157	SLEV	10.8	2015-09-15	El Paso	*Cx. tarsalis*	TX DSHS	SLEV	NO	99.96	III	PromethION P2S
10328	SLEV	12.2	2018-09-27	El Paso	*Cx. tarsalis*	TX DSHS	SLEV	YES	100.00	III	PromethION P2S
10481	SLEV	18.9	2013-10-29	Nueces	*Cx. quinquefasciatus*	TX DSHS	SLEV	YES	99.94	II	PromethION P2S
11229	SLEV	10.9	2017-10-11	El Paso	*Cx. tarsalis*	TX DSHS	SLEV	YES	100.00	III	PromethION P2S
6222	SLEV/ WNV	13.55	2019-08-06	El Paso	*Cx. tarsalis*	TX DSHS	SLEV/WNV	NO	60.95	III	PromethION P2S
6674	SLEV/ WNV	26.48	2019-08-13	El Paso	*Cx. tarsalis*	TX DSHS	SLEV/WNV	YES	100.00	III	PromethION P2S
TXAR96038	SLEV	7.9	2009-08	Jefferson	*Cx. quinquefasciatus*	BEI	SLEV	YES	100.00	II	PromethION P2S

Isolate	Virus	CT Value	Date of Collection	County	Species	Provider	Virus	Sample Used for Analysis	Genome Completeness	SLEV Genotype	Sequencing Platform
V07457	SLEV	7.62	2013-08-09	Harris	*Cx. quinquefasciatus*	BEI	SLEV	YES	100.00	II	PromethION P2S
V08449	SLEV	7.5	2013-08	Harris	*Cx. quinquefasciatus*	BEI	SLEV	YES	99.99	II	PromethION P2S
V08458	SLEV	8.5	2013-08	Harris	*Cx. quinquefasciatus*	BEI	SLEV	YES	100.00	II	PromethION P2S

Appendix Table 3. Genotyped SLEV genomes (not used in phylogenomic analyses)

Pool	Completeness (%)	Top BLASTn Hit	E-value	Percent ID	Genotype
4799	55.06	KT823415.1	0	99.68	III
6029	67.51	MW075085.1	0	99.88	III
6354	9.22	EF158052.1	0	98.88	II
6822	8.7	MN233312.1	0	99.68	III
2400375	24.83	MN233312.1	0	99.74	III
2400450	11.49	MN233329.1	0	99.67	III

Appendix Table 4. SLEV primer pool oligos

Oligo Name	5'-3' Oligo Sequence	Start	Stop	Pool #	Sense
SLEV_olivar_1f	GCGAACAGTTTTTTAGCAGGGA	69	90	1	+
SLEV_olivar_1r	CCATGCACAAAAATTGCAACCTC	1374	1396	1	-
SLEV_olivar_2f	GCAAAAGAGATGTTGTGGACCG	1237	1258	2	+
SLEV_olivar_2r	CTCCTCCACATTTCAATTCACGTC	2494	2517	2	-
SLEV_olivar_3f	GCTTTGGCGACCACATGGAA	2181	2200	1	+
SLEV_olivar_3r	TGGTGTATCGCAGTGGTGG	3420	3440	1	-
SLEV_olivar_4f	ACGAGCCGTCATGGGAGA	3104	3121	2	+
SLEV_olivar_4r	CAATGAAACCGTGCACTCAAGC	4519	4540	2	-
SLEV_olivar_5f	TTGAGAAAGCAGCAGACATCACAT	4363	4386	1	+
SLEV_olivar_5r	CCCTGTGAAGTTGGTGATCCA	5646	5666	1	-
SLEV_olivar_6f	GCCCACTTCATTGATCCAGCA	5454	5474	2	+
SLEV_olivar_6r	TTGCTTCTCAGGTTCTGGAATCA	6802	6824	2	-
SLEV_olivar_7f	CAGGAAAGGAGTTGGTAAAATGGG	6668	6691	1	+
SLEV_olivar_7r	TCTGCATGAGTTGCGGTTC	8004	8022	1	-
SLEV_olivar_8f	AGGAGCCACTCTTGGAGAGA	7682	7701	2	+
SLEV_olivar_8r	CCATTTCCCAGAACTTTGGATCTTC	8955	8979	2	-
SLEV_olivar_9f	TCTCAAAACCATGGGATATGATCACAA	8659	8685	1	+
SLEV_olivar_9r	GTGAGATTCTGGCTCTGCCAAT	9888	9909	1	-
SLEV_olivar_10f	GCCTGATGGGAAAACCTACATGGA	9428	9451	2	+
SLEV_olivar_10r	TCTAACCTCTAGTCCTTACGCCA	10707	10729	2	-

Start and Stop coordinates are based on DQ525916.1

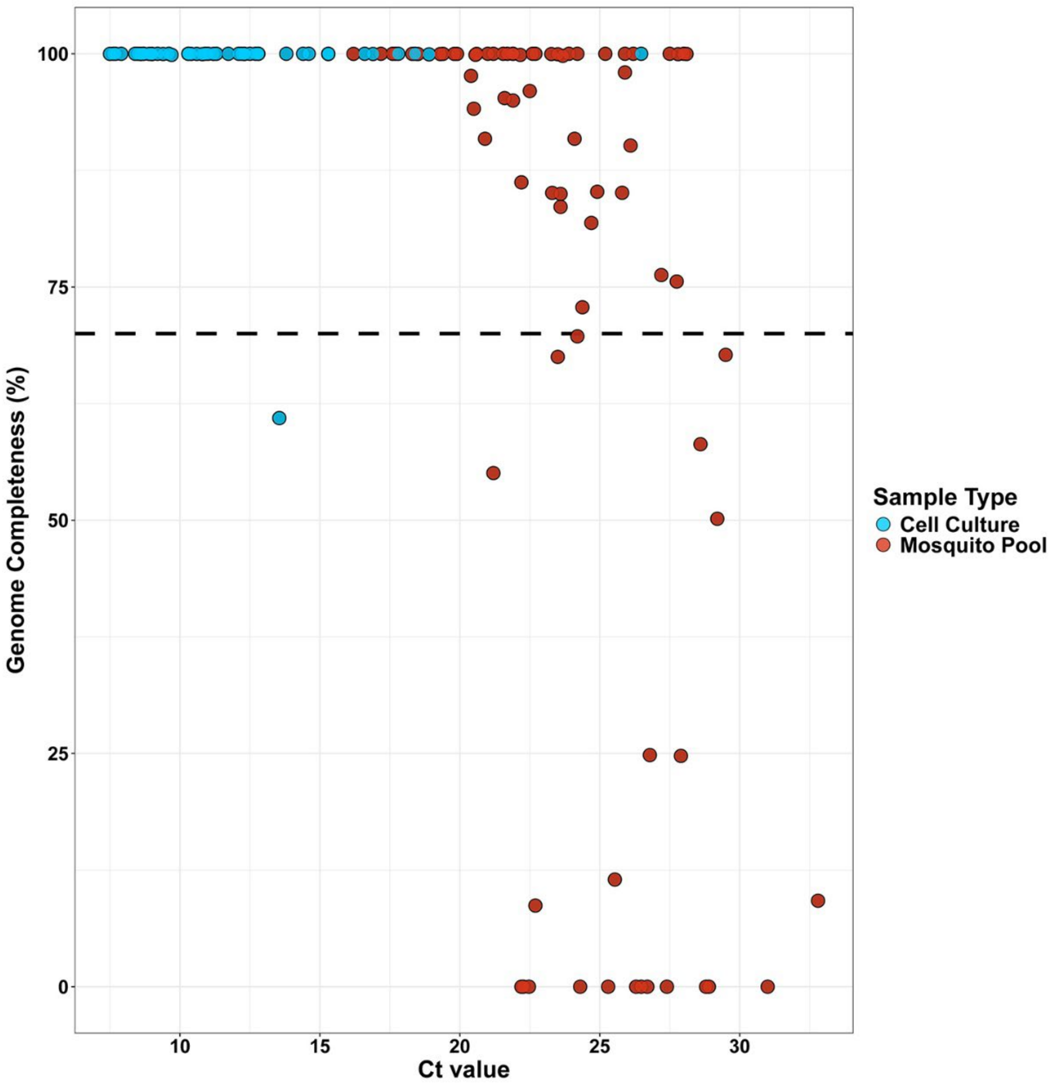

Appendix Figure 1. Sample Genome Completeness and Ct Plot. The amount of the genome recovered from_sequencing (genome completeness) was plotted against the Ct value determined for each sample, either a cell culture sample from viral isolation (blue) or a mosquito pool sample (red). The dotted line indicates 70% genome completeness which we used as the metric for successful sequencing. The median Ct of successful samples was 22.7 (range 16.2-29.5) with 90% of successful samples with a Ct ≤27.4.

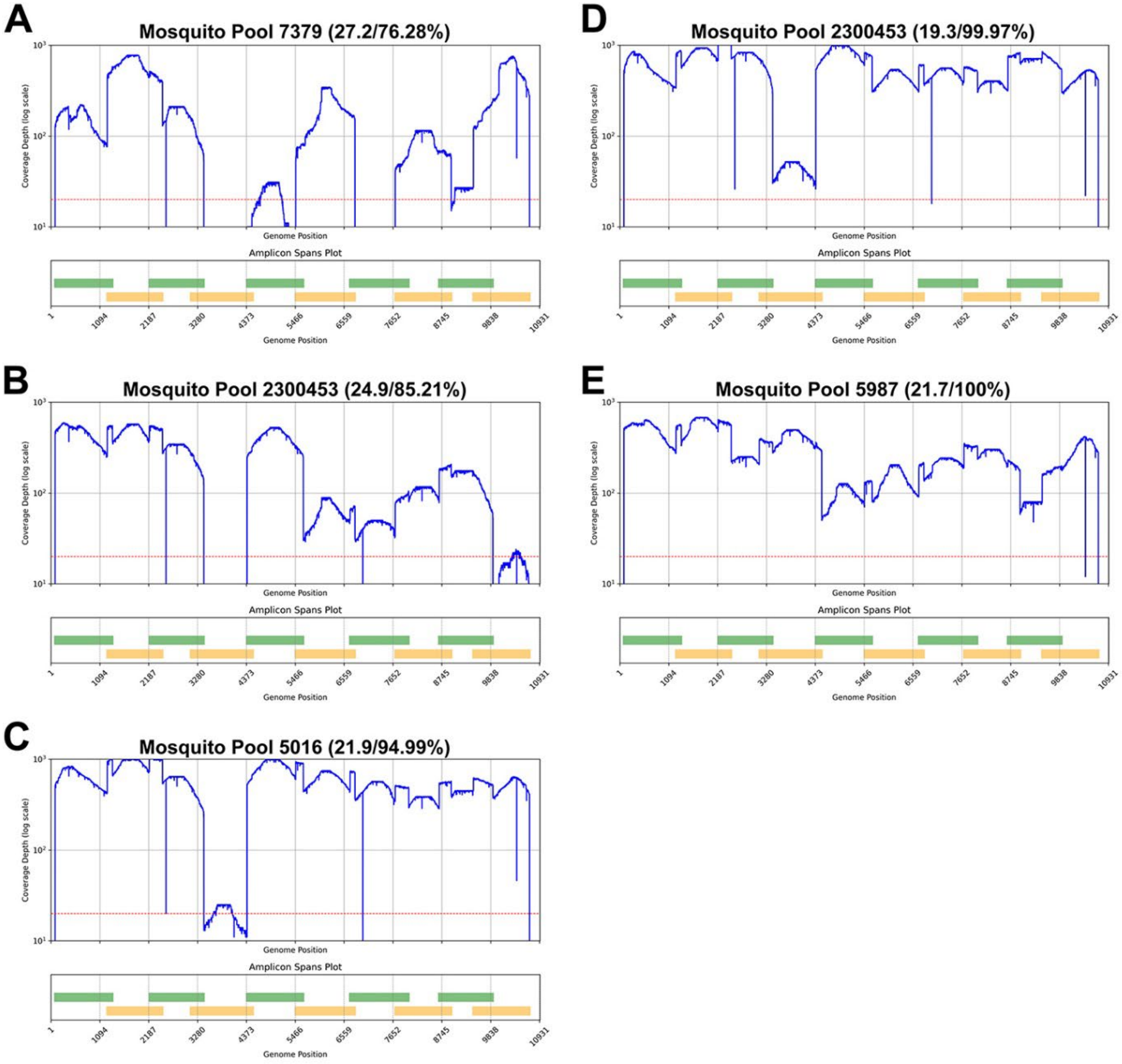

Appendix Figure 2. Genome Read Depth Plots of Representative Samples. The read depths of different samples were plotted to visualize how well the tiled-amplicon primer scheme worked across samples with different Ct values. The read depth is shown on the Y-axis in log scale with the X-axis reflecting base positions on the SLEV reference genome. The solid blue line indicates read depth. The dotted red line indicates 20x read depth which is the threshold for a base being called by ViralRecon using the ARTIC minion pipeline. The “Amplicon Spans Plot” indicates the positions of the amplicons along the SLEV genome with green and yellow spans indicating pool 1 and pool 2 amplicons, respectively. The values in parentheses by the sample name indicate the sample’s Ct value and the genome completeness, respectively.

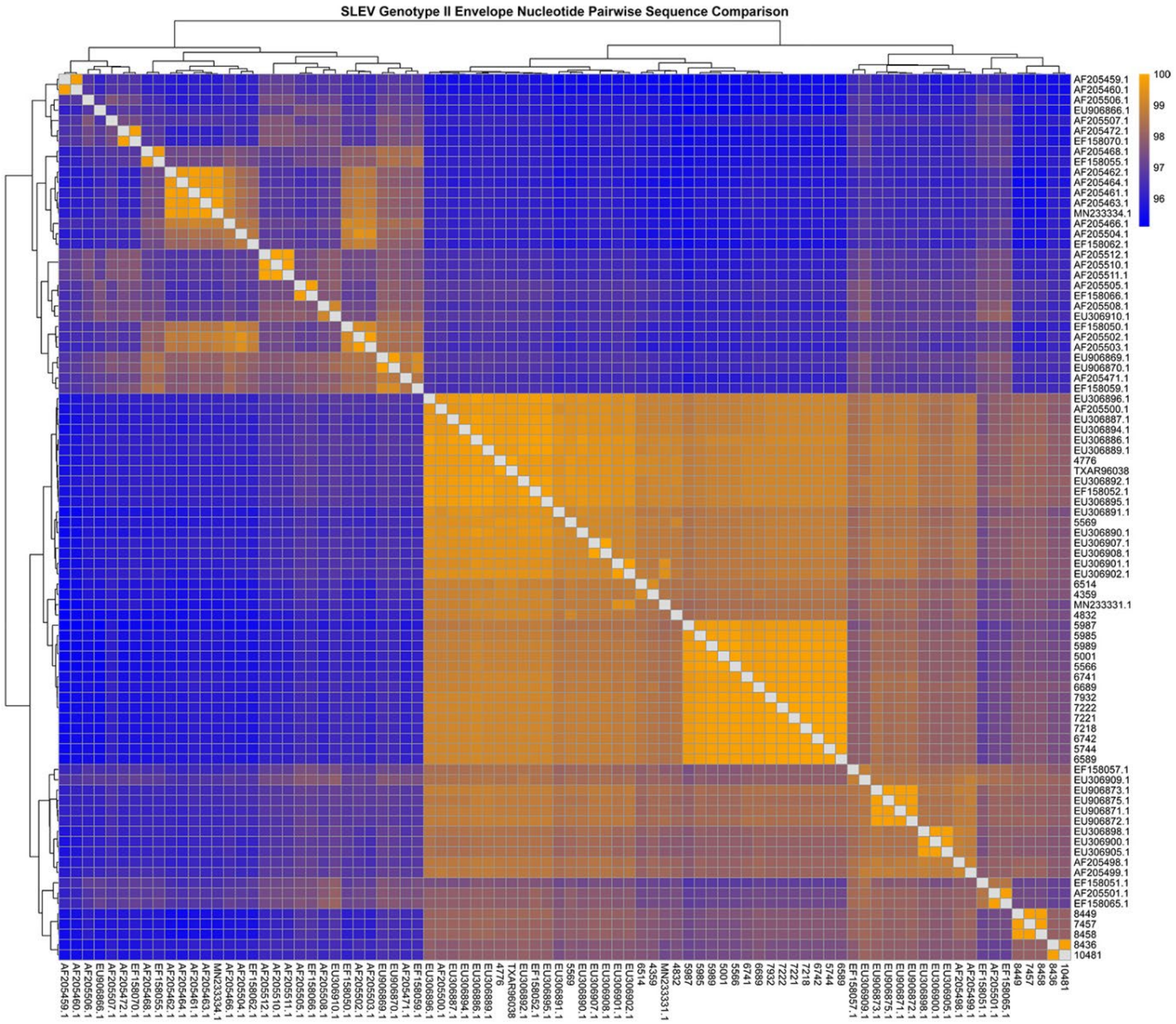

Appendix Figure 3. Heatmap of SLEV Genotype II Envelope Gene Sequence Similarities. The pairwise nucleotide sequence similarities of 86 SLEV genotype II envelope genes were determined and plotted in a heatmap scaling from blue (more divergent) to orange (more similar). The subclades within genotype II are indicated on the bottom of the figure. The top and left of the figure are the hierarchical clustering of columns and rows respectively as determined by the pheatmap program.

References

1. Darsie R, Jr, Ward RA. Identification and geographical distribution of the mosquitoes of North America, north of Mexico. Gainesville (Florida): University Press of Florida; 2005.

2. Lambert AJ, Martin DA, Lanciotti RS. Detection of North American eastern and western equine encephalitis viruses by nucleic acid amplification assays. J Clin Microbiol. 2003;41:379–85. PubMed https://doi.org/10.1128/JCM.41.1.379-385.2003

3. Brault AC, Fang Y, Reisen WK. Multiplex qRT-PCR for the detection of western equine encephalomyelitis, St. Louis encephalitis, and West Nile viral RNA in mosquito pools (Diptera: Culicidae). J Med Entomol. 2015;52:491–9. PubMed https://doi.org/10.1093/jme/tjv021

4. Burkett-Cadena ND. Mosquitoes of the southeastern United States. Tuscaloosa (Alabama): University of Alabama Press; 2013.

5. Tsai TF, Cobb WB, Bolin RA, Gilman NJ, Smith GC, Bailey RE, et al. Epidemiologic aspects of a St. Louis encephalitis outbreak in Mesa County, Colorado. Am J Epidemiol. 1987;126:460–73. PubMed https://doi.org/10.1093/oxfordjournals.aje.a114677

6. Kneubehl AR. Texas-SLEV-genome-surveillance. 2025 [cited 2025 Jan 1]. https://github.com/kneubehl/Texas-SLEV-Genome-Surveillance

7. Lanciotti RS, Kerst AJ. Nucleic acid sequence-based amplification assays for rapid detection of West Nile and St. Louis encephalitis viruses. J Clin Microbiol. 2001;39:4506–13. PubMed https://doi.org/10.1128/JCM.39.12.4506-4513.2001

8. Wang MX, Lou EG, Sapoval N, Kim E, Kalvapalle P, Kille B, et al. Olivar: towards automated variant aware primer design for multiplex tiled amplicon sequencing of pathogens. Nat Commun. 2024;15:6306. PubMed https://doi.org/10.1038/s41467-024-49957-9

9. Fu L, Niu B, Zhu Z, Wu S, Li W. CD-HIT: accelerated for clustering the next-generation sequencing data. Bioinformatics. 2012;28:3150–2. PubMed https://doi.org/10.1093/bioinformatics/bts565

10. Katoh K, Standley DM. MAFFT multiple sequence alignment software version 7: improvements in performance and usability. Mol Biol Evol. 2013;30:772–80. PubMed https://doi.org/10.1093/molbev/mst010

11. Minh BQ, Schmidt HA, Chernomor O, Schrempf D, Woodhams MD, von Haeseler A, et al. IQ-TREE 2: new models and efficient methods for phylogenetic inference in the genomic era. Mol Biol Evol. 2020;37:1530–4. PubMed https://doi.org/10.1093/molbev/msaa015

12. Hoang DT, Chernomor O, von Haeseler A, Minh BQ, Vinh LS. UFBoot2: improving the ultrafast bootstrap approximation. Mol Biol Evol. 2018;35:518–22. PubMed https://doi.org/10.1093/molbev/msx281

13. Chernomor O, von Haeseler A, Minh BQ. Terrace aware data structure for phylogenomic inference from supermatrices. Syst Biol. 2016;65:997–1008. PubMed https://doi.org/10.1093/sysbio/syw037

14. Letunic I, Bork P. Interactive Tree of Life (iTOL) v6: recent updates to the phylogenetic tree display and annotation tool. Nucleic Acids Res. 2024;52(W1):W78–82. PubMed https://doi.org/10.1093/nar/gkae268

15. Camacho C, Coulouris G, Avagyan V, Ma N, Papadopoulos J, Bealer K, et al. BLAST+: architecture and applications. BMC Bioinformatics. 2009;10:421. PubMed https://doi.org/10.1186/1471-2105-10-421

16. Patel H, Monzón S, Varona S, Espinosa-Carrasco J, Garcia M, Heuer M, et al. nf-core/viralrecon: nf-core/viralrecon v2. 6.0-Rhodium Raccoon. 2023 [cited 2025 Jan 1]. https://zenodo.org/records/7764938

17. Kalyaanamoorthy S, Minh BQ, Wong TKF, von Haeseler A, Jermiin LS. ModelFinder: fast model selection for accurate phylogenetic estimates. Nat Methods. 2017;14:587–9. PubMed https://doi.org/10.1038/nmeth.4285

18. Yu G, Smith DK, Zhu H, Guan Y, Lam TTY. ggtree: an R package for visualization and annotation of phylogenetic trees with their covariates and other associated data. Methods Ecol Evol. 2017;8:28–36. https://doi.org/10.1111/2041-210X.12628

19. Rambaut A, Lam TT, Max Carvalho L, Pybus OG. Exploring the temporal structure of heterochronous sequences using TempEst (formerly Path-O-Gen). Virus Evol. 2016;2:vew007. PubMed https://doi.org/10.1093/ve/vew007

20. Drummond AJ, Rambaut A. BEAST: Bayesian evolutionary analysis by sampling trees. BMC Evol Biol. 2007;7:214. PubMed https://doi.org/10.1186/1471-2148-7-214
